# Supplementary material for: Designing microbial communities to maximize the thermodynamic driving force for the production of chemicals
Source: PLoS Comput Biol. 2021 Jun 15;17(6):e1009093. doi: 10.1371/journal.pcbi.1009093 (PMC8232427; doi:10.1371/journal.pcbi.1009093)
Supplement: S1 Text — (PDF) [file pcbi.1009093.s001.pdf]

Designing Microbial Communities to Maximize the Thermodynamic Driving Force for the Production of Chemicals

S1 Text

Pavlos Stephanos Bekiaris[1] and Steffen Klamt[2,*]

[1]Max Planck Institute for Dynamics of Complex Technical Systems, Magdeburg, Germany

[2]Max Planck Institute for Dynamics of Complex Technical Systems, Magdeburg, Germany

* klamt@mpi-magdeburg.de

Proof showing that division of labor (DoL) cannot increase the product synthesis flux compared to a single-strain solution for arbitrary pathways and kinetics (under assumption of constant metabolite concentrations in the strains)

We assume we are given a pathway from a substrate S to a product P consisting of q reactions (each catalyzed by an associated enzyme E_i) and m metabolites (also including (e.g. allosteric) effectors of the enzymes of this pathway). The reaction rate (unit: [mmol/(gDW h)] of a reaction i is denoted by r_i (and the vector of all reaction rates by $\mathbf{r}$) and the concentration of a metabolite k by c_k (vector of all metabolite concentrations: $\mathbf{c}$). For any given steady-state rate vector $\mathbf{r}$ of the pathway we can scale it to $\tilde{\mathbf{r}}$ such that the rate of the (last) reaction producing the product P is unity: $\tilde{r}_p = 1$.

Each reaction has an associated kinetic rate law in the most general form: $r_i = [E_i] \cdot k_{cat,i} \cdot f_i(\mathbf{c})$ and the enzyme costs for having $\tilde{r}_P = 1$ are then $\sum_i [E_i] = \sum_i \tilde{r}_i / (k_{cat,i}\, f_i(\boldsymbol{c}))$ (1)

Therefore, under a given maximal amount of resources ($\sum_i [E_i] \leq R$, R with unit [g enzyme / gDW]), the maximal rate r_P we can get is

$$r_P = \frac{R}{\sum_i \tilde{r}_i / (k_{cat,i}\, f_i(\mathbf{c}))} \qquad (2)$$

Given a total amount of biomass B (in [gDW]), the absolute maximal production flux J_P (unit: [mmol/h]) with the single strain is

$$J_P = B r_P = B \frac{R}{\sum_i \tilde{r}_i / (k_{cat,i}\, f_i(\mathbf{c}))} \qquad (3)$$

In a community, the pathway can be split, e.g., into two reactions sets (strains) called "1" and "2" with q_1 and q_2 reactions, respectively, such that $q_1 + q_2 = q$. The overall steady-state rate vector $\mathbf{r}^{\boldsymbol{c}}$ over the community can be written as

$$\mathbf{r}^{\boldsymbol{c}} = \mathbf{r}^1 + \mathbf{r}^2 \qquad (4)$$

(where the rates of the reactions of strain 2 (1) are zero in $\mathbf{r}^1$ ($\mathbf{r}^2$)). Importantly, in the community, additional transport reactions are needed for the exchange of intermediate metabolites (one reaction in each strain for each intermediate exchanged). In the following we will not include these reactions in our calculations (i.e., we assume zero enzyme costs for them and do not include their rates in the community rate vector $\mathbf{r}^{\boldsymbol{c}}$), but we keep in mind that they will usually enhance the overall enzyme costs and thus reduce the possible maximal flux in the community. With $\tilde{\mathbf{r}}^1$ and $\tilde{\mathbf{r}}^2$, we denote again the normalized reaction rate vectors of the two strains, such that in the normalized overall community flux vector

$\tilde{\mathbf{r}}^{\boldsymbol{c}} = \tilde{\mathbf{r}}^1 + \tilde{\mathbf{r}}^2$ (5)

we get a product synthesis flux of $\tilde{r}_p^c = 1$.

As in the main text, in the following we assume that all metabolite concentrations in the two strains remain the same as in the single strain and that the maximal enzyme concentration in each strain is again limited

by R. Similar as we did above for eq. (2), the maximal possible fluxes per gram of biomass in the two strains reads

$$\alpha^1 = \frac{R}{\sum_i \tilde{r}_i^1 / (k_{cat,i}\, f_i(\mathbf{c}))} \qquad (6)$$

$$\alpha^2 = \frac{R}{\sum_i \tilde{r}_i^2 / (k_{cat,i}\, f_i(\mathbf{c}))} \qquad (7)$$

where α^1 (α^2) denote the number of times that $\tilde{\mathbf{r}}^1$ ($\tilde{\mathbf{r}}^2$) can run per gram of biomass of strain 1 (strain 2). Here, depending on the enzyme cost for each reaction and how we divided the pathway on the two strains, α^1 and α^2 will usually not be equal. However, we need to add $\tilde{\mathbf{r}}^1$ and $\tilde{\mathbf{r}}^2$ in equal amounts to get $\tilde{r}_P = 1$. This can be achieved by partitioning the biomasses of the two strains, B^1 and B^2 such that the resulting total fluxes in the two strains match each other:

$$B^1\alpha^1 = B^2\alpha^2 = J_P \qquad (8)$$

The sum of the two biomasses is, as for the single strain solution, again limited by the total available investment in biomass B, i.e. $B = B^1 + B^2$. With that we derive $(B - B^2)\alpha^1 = B^2\alpha^2$ and thus $B^2 = \alpha^1 B / (\alpha^2 + \alpha^1)$. For the total (product synthesis) flux in the community we then get

$$J_P = B^1\alpha^1 = B^2\alpha^2 = \alpha^1\alpha^2 B / (\alpha^2 + \alpha^1) = B / (1/\alpha^1 + 1/\alpha^2) \qquad (9)$$

Substituting α^1 and α^2 in (9) with the expressions (6) and (7) and taking (5) into account we get

$$J_P = B \frac{R}{\sum_i \tilde{r}_i^1 / (k_{cat,i}\, f_i(\mathbf{c})) + \sum_i \tilde{r}_i^2 / (k_{cat,i}\, f_i(\mathbf{c}))} = \frac{R}{\sum_i \tilde{r}_i^c / (k_{cat,i}\, f_i(\mathbf{c}))} \qquad (10)$$

Thus, the derived solution for the maximal total product flux in the DoL community (10) is essentially the same as for the found single strain solution (3), hence, DoL brings no advantage.

Again, as stated above, the total product flux J_P in the community may decrease due to possible transport costs (for example, some extra ATP might then be required whose production will reduce the available enzyme resources R for the product pathway).

If the metabolite concentrations would be allowed to be different in the two strains, the total product flux may decrease or increase, depending on the saturation functions f_i (see also the last paragraph of the Discussion section in the main manuscript).
