# Supplementary material for: Designing microbial communities to maximize the thermodynamic driving force for the production of chemicals
Source: PLoS Comput Biol. 2021 Jun 15;17(6):e1009093. doi: 10.1371/journal.pcbi.1009093 (PMC8232427; doi:10.1371/journal.pcbi.1009093)
Supplement: S2 Text — (PDF) [file pcbi.1009093.s002.pdf]

# Designing Microbial Communities to Maximize the Thermodynamic Driving Force for the Production of Chemicals

## S2 Text

Pavlos Stephanos Bekiaris[1] and Steffen Klamt[2,*]

[1]Max Planck Institute for Dynamics of Complex Technical Systems, Magdeburg, Germany

[2]Max Planck Institute for Dynamics of Complex Technical Systems, Magdeburg, Germany

* klamt@mpi-magdeburg.de

## Detailed description of configuration of the single strain models and the derived (multi-strain) community models of *E. coli*

In the main text, ASTHERISC was exemplarily applied to analyze single-species (multi-strain) community models of *E. coli,* which were created with CommModelPy and then analyzed with the ASTHERISC package. In total, we considered three different community models. Two of them, *ecolicore2double* and *ecolicore2triple*, are based on the *EColiCore2* model [1] (a reduced version of the genome-scale model *i*JO1366 [2] containing 499 reactions and 486 metabolites). *ecolicore2double* contains two and *ecolicore2triple* three duplicates of the *EColiCore2* network as strains in the community model. The third community model, *iML1515double*, contains two copies of the recently published genome-scale network *i*ML1515 [3]. The input models as well as the derived community models use reaction and metabolite identifiers in accordance with the BiGG Models database [4] and the three community models can be found as SBML models in the respective GitHub repositories, together with the CommModelPy-based scripts used to create them.

The CommModelPy repository can be found under the following link:

https://github.com/klamt-lab/CommModelPy

The ASTHERISC package can be found under the following link:
Project home page: https://github.com/klamt-lab/autoastherisc

In the following we give a detailed description of the assignment/computation of $\Delta_r G'^0$ values, the assembly of the community models and the used constraints for the calculations.

1) As a first step, we sought to assign a $\Delta_r G'^0$ value to each reaction in the core as well as in the genome-scale (single-species) models. As source for $\Delta_r G'^0$, we used the Python-based eQuilibrator API, which uses the eQuilibrator component contribution method [5]. In order to work with this API, in a preprocessing step, a mapping from BiGG metabolite identifiers to MetaNetX [6] identifiers was generated for both *EcoliCore2* and *i*Ml1515. With this mapping, the reactions of the models were translated to reaction strings using the respective MetaNetX metabolite identifiers. Using these strings, the eQuilibrator API calculated the $\Delta_r G'^0$ values for each reaction with the standard settings of a temperature of 298.15 K, an ionic strength of 0.25 M, a pH of 7.0 and a pMg of 3.0. The calculated $\Delta_r G'^0$ uncertainties were not used for the subsequent reactions, i.e., an uncertainty of 0 kJ/mol was assumed to prevent thermodynamically infeasible solutions (e.g. non-zero fluxes in a cycle which may become possible when choosing all $\Delta_r G'^0$ at the lower end of their feasible range). For most reactions where no $\Delta_r G'^0$ could be found or determined at all or only with an uncertainty greater than 10000 kJ/mol (which is an indicator for a calculation problem), the value NaN was assigned and these reactions were not allowed to take part in the calculations (i.e., upper and lower bound of those reactions were set to zero). The only exception from this are reactions whose BiGG identifier ended with “tpp”, “t1pp” or “tex” since these endings stand for diffusion or facilitated (e.g. symporter-assisted) transport processes. For these important reactions, the $\Delta_r G'^0$ was left unconstrained but the reaction not blocked because the precise transport mechanisms are often not known. For the important oxidative phosphorylation associated membrane-bound reactions of the ATP synthase (BiGG identifier ATPS4), the NADH dehydrogenases (NADH16, NADH17 and NADH18) and the cytochrome oxidases (CYTBDpp, CYTBD2pp and CYTBO3_4pp), a correction factor was applied to the $\Delta_r G'^0$ values calculated by the eQuilibrator API in order to correct for their presence in the membrane and their associated proton transport. The correction factor $C_j$ for reaction $j$ is as follows (as given in eq. (9) in [7], which is based on the formulation in [8]):

$C_j = c_j F\Delta\Psi - 2.3 h_j RT\Delta pH$ (24)

where $c_j$ stands for the caused charge difference between the outside and the inside compartment, $h_j$ for the number of transported protons, $\Delta\Psi$ for the electrochemical potential of the membrane, $F$ for the Faraday constant, $R$ for the gas constant, $T$ for the temperature and $\Delta pH$ for the pH difference between the affected compartments. In this study, as in [7], $\Delta\Psi$ was assumed to be -130 mV and $\Delta pH$ to be 0.4. $T$ was set to 298.15 K in compliance with the $\Delta_r G'^0$ calculated by the eQuilibrator API. For the values of $c_j$ and $h_j$, the same values as in the supplementary data of [7] were used. The complete $\Delta_r G'^0$ dataset, as well as the scripts generating it, can be found as JSON in the GitHub repository of CommModelPy.

As a result, in the base models *EColiCore2* and *iML1515* used for the community models, around 18% of the reactions in EColiCore2 and 24% of the reactions in iML1515 were blocked due to a lack of $\Delta_r G'^0$ values (which also reduced the number of producible target metabolites to 161 in the two *EColiCore2*-related community models and 254 in *iML1515*double; see also main text).

2) As described in the main manuscript, in the next step we added to each metabolite (in the single species model), that occurs in the periplasmic or cytoplasmic compartment, an exchange reaction (if a metabolite occurs in both compartments, the periplasmic representation of that metabolite is used). When the single-strain models are combined to the community model, these “extra exchange reaction” will allow export and import of compounds from/to the exchange compartment and thus exchange of metabolites between different strains. Furthermore, if one of these compounds is considered as the desired final product (the target metabolite), an additional exchange will be added from the exchange compartment to the environment thus allowing accumulation of the compound in the medium (and thus its production). Importantly, apart from the “extra metabolite exchanges”, the original (single) species models contain “standard exchanges” (e.g. for substrate uptake or product excretion), which are also available in the community model and allow the strains in the community to import the substrate and nutrients from the environment and to release therein typical products such as $CO_2$ or fermentation products such as acetate, ethanol etc. (hence, in the community model, those compounds can be exchanged between the cell and the exchange compartment and then imported/exported from/to the environment. In addition, metabolites which occurred in “demand” reactions where these metabolites simply vanish in the environment were added as standard output metabolites. A list of the associated standard exchange metabolites for the two models are given in Table A.

In all models, D-glucose served as carbon source and its uptake rate was fixed to 1 mmol/gDW/h in the calculations. For nearly all metabolite exchange reactions between the organisms and the exchange compartment, a $\Delta_r G'^0$ of 0 is assumed. Although this represents a simplification, this still enforces a concentration gradient between the compartments in order to thermodynamically allow a metabolite exchange. The only exceptions are the exchange reactions for water (h2o) and protons (h) for which no Gibbs free energy was set since water is assumed to be ubiquitous and since the proton gradient between the intracellular compartments is not directly modeled here.

With all added exchange reactions, the total size of the three models was at follows: 2211 reactions and 1398 metabolites (*ecolicore2double*), 3105 reactions and 1885 metabolites (*ecolicore2triple*) and 8265 reactions and 4924 metabolites (*iML1515double*).

Table A. List of standard exchange metabolites used in the community models *ecolicore2double, ecolicore2triple* and *iML1515double*. “p“ stands for periplasm and “c“ for cytoplasm (exchange of cytoplasmic metabolites occurs with “demand“ reactions).

| Metabolite ID | Common metabolite name | Can be secreted? | Can be imported? |
|---|---|---|---|
| 4crsol_c | P-cresol | Yes | No |
| 5drib_c | 5'-deoxyribose | Yes | No |
| aacald_c | Aminoacetaldehyde | Yes | No |
| ac_p | Acetate | Yes | No |
| amob_c | S-adenosyl-4-methylthio-2-oxobutanoate | Yes | No |
| ca2_p | Calcium 2+ | No | Yes |
| cl_p | Chloride | No | Yes |
| co2_p | Carbon dioxyde | Yes | Yes |
| cobalt2_p | Cobalt 2+ | No | Yes |
| cu2_p | Copper 2+ | No | Yes |
| etoh_p | Ethanol | Yes | No |
| fe2_p | Iron 2+ | No | Yes |
| fe3_p | Iron 3+ | No | Yes |
| for_p | Formate | Yes | No |
| glc__D_p | D-glucose | No | Yes |
| h_p | Proton | Yes | Yes |
| h2o_p | Water | Yes | Yes |
| k_p | Potassium | No | Yes |
| lac__D_p | D-lactose | Yes | No |
| meoh_p | Methanol | Yes | No |
| mg2_p | Magnesium 2+ | No | Yes |
| mn2_p | Manganese 2+ | No | Yes |
| mobd_p | Molybdate | No | Yes |
| mththf_c | (2R,4S)-2-methyl-2,3,3,4-tetrahydroxytetrahydrofuran | Yes | No |
| na1_p | Sodium (in iML1515 only) | No | Yes |
| ni2_p | Nickel 2+ | No | Yes |
| nh4_p | Ammonium | No | Yes |
| o2_p | Oxygen | No | Yes |
| oxam_c | Oxamate | Yes | No |
| pi_p | Phosphate | No | Yes |
| sel_p | Selenate (in iML1515 only) | No | Yes |
| slnt_p | Selenite (in iML1515 only) | No | Yes |
| so4_p | Sulfate | No | Yes |
| succ_p | Succinate | Yes | No |
| tungs_p | Tungsten (in iML1515 only) | No | Yes |
| zn2_p | Zinc 2+ | No | Yes |

3) To focus entirely on product synthesis, in all community models, the growth rate and the minimal flux of nongrowth-associated ATP maintenance (ATPM) were set to 0.

4) As in the original OptMDFpathway study [9], the standard intracellular metabolite concentration range was set from 1 µM to 20 mM. The standard metabolite concentration range in the exchange compartment was set from 1 µM to 10 M as the extracellular space does not pose strict constraints on the solubility of the metabolites. For water (h2o) and protons (h), the concentration was fixed to 1M in all compartments, since the used $\Delta_r G'^0$ values from the eQuilibrator API already assume this "active" concentration for water and are based on the assumption of no free energy in protons. In addition, several cytoplasmic metabolite concentration ratios were fixed: ATP:ADP in a ratio range from 3 to 10, ADP:AMP from 0.5 to 2, NAD+:NADH from 3 to 10 and NADPH:NADP from 3 to 10. Furthermore, due to its weak solubility range in intracellular medium, the concentration of $CO_2$ (periplasmic and cytosolic) was restricted to values between 1 µM and 1 mM.

5) A minimal MDF advantage of 0.2 kJ/mol for a community compared to a single-organism solution was demanded. If the advantage is lower than 0.2 kJ/mol, the solution is ignored. This excludes solutions with just marginal advantages. The minimal MDF which has to be reached by a community was set to 0 kJ/mol, this excludes all thermodynamically infeasible solutions with an MDF smaller than 0 kJ/mol but includes all solutions where the community is able to provide a thermodynamically feasible pathway in a case which would be infeasible in a single strain only. The minimal value for a reaction flux in order to be considered as the flux of an active reaction was set to $10^{-6}$ mmol/gDW/h in order to prevent numeric problems. The precision for ASTHERISC's MDF and maximal yield approximation routines were set to 0.1 kJ/mol and 0.01 mmol/mmol, respectively. For all MILPs, a maximal run time of 1000 s was set. If a MILP was due to take longer, it was aborted and the solution was set as inconclusive, and a warning was given in the final text reports.

## References


1. Hädicke O, Klamt S. EColiCore2: a reference network model of the central metabolism of Escherichia coli and relationships to its genome-scale parent model. Scientific Reports. 2017;7:39647. doi: 10.1038/srep39647.
2. Orth JD, Conrad TM, Na J, Lerman JA, Nam H, Feist AM, et al. A comprehensive genome-scale reconstruction of Escherichia coli metabolism—2011. Molecular Systems Biology. 2011;7(1):535. doi: 10.1038/msb.2011.65.
3. Monk JM, Lloyd CJ, Brunk E, Mih N, Sastry A, King Z, et al. i ML1515, a knowledgebase that computes Escherichia coli traits. Nature Biotechnology. 2017;35(10):904-8. doi: 10.1038/nbt.3956.
4. King ZA, Lu J, Dräger A, Miller P, Federowicz S, Lerman JA, et al. BiGG Models: A platform for integrating, standardizing and sharing genome-scale models. Nucleic Acids Research. 2016;44(D1):D515-D22. doi: 10.1093/nar/gkv1049.
5. Flamholz A, Noor E, Bar-Even A, Milo R. eQuilibrator—the biochemical thermodynamics calculator. Nucleic Acids Research. 2012;40(D1):D770-D5. doi: 10.1093/nar/gkr874.
6. Ganter M, Bernard T, Moretti S, Stelling J, Pagni M. MetaNetX.org: a website and repository for accessing, analysing and manipulating metabolic networks. Bioinformatics. 2013;29(6):815-6. doi: 10.1093/bioinformatics/btt036.
7. Hamilton Joshua J, Dwivedi V, Reed Jennifer L. Quantitative Assessment of Thermodynamic Constraints on the Solution Space of Genome-Scale Metabolic Models. Biophysical Journal. 2013;105(2):512-22. doi: 10.1016/j.bpj.2013.06.011.
8. Henry CS, Broadbelt LJ, Hatzimanikatis V. Thermodynamics-Based Metabolic Flux Analysis. Biophysical Journal. 2007;92(5):1792-805. doi: 10.1529/biophysj.106.093138.
9. Hädicke O, Kamp Av, Aydogan T, Klamt S. OptMDFpathway: Identification of metabolic pathways with maximal thermodynamic driving force and its application for analyzing the endogenous CO2 fixation potential of Escherichia coli. PLOS Computational Biology. 2018;14(9):e1006492. doi: 10.1371/journal.pcbi.1006492.
