# Supplementary material for: Designing microbial communities to maximize the thermodynamic driving force for the production of chemicals
Source: PLoS Comput Biol. 2021 Jun 15;17(6):e1009093. doi: 10.1371/journal.pcbi.1009093 (PMC8232427; doi:10.1371/journal.pcbi.1009093)
Supplement: S3 Text — (PDF) [file pcbi.1009093.s003.pdf]

Designing Microbial Communities to Maximize the Thermodynamic Driving Force for the Production of Chemicals

S3 Text

Pavlos Stephanos Bekiaris[1] and Steffen Klamt[2,*]

[1]Max Planck Institute for Dynamics of Complex Technical Systems, Magdeburg, Germany

[2]Max Planck Institute for Dynamics of Complex Technical Systems, Magdeburg, Germany

* klamt@mpi-magdeburg.de

ASTHERISC report for the toy model

The following text shows the ASTHERISC report generated for the community solution of the toy model (see Fig. 2 and Results section in the main text). The corresponding file can also be found in the ASTHERISC package's GitHub repository.

```
==TARGET WITH COMMUNITY ADVANTAGE: R_EX_C_P_exchg==
Optimal yield without community and with deactivated dG0=NaN reactions without any
MDF constraint [in mol/mol]: 1
Optimal yield without community without any MDF constraint [in mol/mol]: 1
Optimal MDF without community with minimal necessary yield as minimal yield
constraint [in kJ/mol]: -0.19137
Reached yield without community at optimal MDF solution (this yield is a lower bound)
[in mol/mol]: 1
Optimal MDF with community with minimal necessary yield as minimal yield constraint
(if no warning given) [in kJ/mol]: 0.46467
Approximated optimal yield with community at maximal community MDF (if no warning
given) [in mol/mol]: 1
Number of active metabolites: 11

Number of active reactions | Total absolute flux per species [in mmol/(gDW*h)]; Both
with minimal absolute flux sum solution and substrate uptake scaled to maximum:
strain1: 2 | 2
strain2: 2 | 2

Extra exchanges and scaled flux [in mmol/(gDW*h)] with minimal absolute flux solution
(negative flux means uptake to species, positive flux secretion from species)
and associated metabolites with concentrations in M:
R_EXCHG_strain1_B_c_to_B | SCALED FLUX: 1 | M_B_c_strain1 [8.5268;8.5308] | M_B_exchg
[7.0694;7.0727]
R_EXCHG_strain2_B_c_to_B | SCALED FLUX: -1 | M_B_c_strain2 [5.8611;5.8639] |
M_B_exchg [7.0694;7.0727]

Metabolites with non-overlapping concentration ranges between species [concentrations
are given in M]:
M_X_c_strain1 [1;1.3689] & M_X_c_strain2 [3.6525;10]
M_B_c_strain1 [8.5268;8.5308] & M_B_c_strain2 [5.8611;5.8639]
M_S_c_strain1 [8.2869;8.2908] & M_S_c_strain2 [unused]
M_C_c_strain1 [unused] & M_C_c_strain2 [3.6525;10]
M_P_c_strain1 [unused] & M_P_c_strain2 [6.0308;6.0336]

Bottleneck reactions (driving force=OptMDF) of single-species solution as calculated
back from community solution:
MDF in single species with reactions occurring in community solution [in kJ/mol]: -
0.19137 (OptMDF in community was 0.46467 kJ/mol, difference is 0.65605 kJ/mol)
>Bottleneck reactions (driving force=OptMDF):
R_S_to_A_strain1 | 1 M_S_c_strain1 -> 1 M_A_c_strain1 + 1 M_X_c_strain1
R_C_to_P_strain1 | 1 M_X_c_strain1 + 1 M_C_c_strain1 -> 1 M_P_c_strain1
R_EXCHG_strain1_P_c_to_P | 1 M_P_c_strain1 (<)-> 1 M_P_exchg
>Connecting metabolites:
R_S_to_A_strain1-> R_C_to_P_strain1; M_X_c_strain1
R_C_to_P_strain1-> R_EXCHG_strain1_P_c_to_P; M_P_c_strain1
```

```
>Connecting metabolite concentration ranges [in M]:
~M_X_c_strain1~
In single species:
[2.1514;2.1514] (range 4.9829e-05)
In community:
M_X_c_strain1: [1;1.3689] (range 0.36894)
M_X_c_strain2: [3.6525;10] (range 6.3475)
~M_P_c_strain1~
In single species:
[4.6285;4.6286] (range 0.0001072)
In community:
M_P_c_strain1: [unused] (range 0)
M_P_c_strain2: [6.0308;6.0336] (range 0.0028514)

Detailed reaction-wise solution reports:
~Detailed solution of community solution~
Active reactions associated with species strain1 (Reaction ID; Flux [in
mmol/(gDW*h)]; Direction; dG0 (adjusted to direction) [in kJ/mol]; dG [in kJ/mol];
Reaction string (adjusted to direction):
S_to_A_strain1; 1; forward; 4; -0.46467; 1 M_S_c_strain1 -> 1 M_A_c_strain1 + 1
M_X_c_strain1
A_to_B_strain1; 1; forward; -5; -0.46548; 1 M_A_c_strain1 + 1 M_X_c_strain1 -> 1
M_B_c_strain1
EXCHG_strain1_S_c_to_S; -1; reverse; 0; -0.46467; 1 M_S_exchg (<)-> 1 M_S_c_strain1
EXCHG_strain1_B_c_to_B; 1; forward; 0; -0.46467; 1 M_B_c_strain1 -> 1 M_B_exchg
Active reactions associated with species strain2 (Reaction ID; Flux [in
mmol/(gDW*h)]; Direction; dG0 (adjusted to direction) [in kJ/mol]; dG [in kJ/mol];
Reaction string (adjusted to direction):
B_to_C_strain2; 1; forward; -5; -0.46467; 1 M_B_c_strain2 -> 1 M_X_c_strain2 + 1
M_C_c_strain2
C_to_P_strain2; 1; forward; 4; -0.46467; 1 M_X_c_strain2 + 1 M_C_c_strain2 -> 1
M_P_c_strain2
EXCHG_strain2_P_c_to_P; 1; forward; 0; -0.46467; 1 M_P_c_strain2 (<)-> 1 M_P_exchg
EXCHG_strain2_B_c_to_B; -1; reverse; 0; -0.46467; 1 M_B_exchg -> 1 M_B_c_strain2
Active reactions of exchange compartment<->environment exchange reactions:
EX_C_S_exchg; -1; reverse; NaN; NaN; (<)-> 1 M_S_exchg
EX_C_P_exchg; 1; forward; NaN; NaN; 1 M_P_exchg ->

~Detailed solution of single-strain solution (recalculated back from community
solution reactions)~
Active reactions associated with species strain1 (Reaction ID; Flux [in
mmol/(gDW*h)]; Direction; dG0 (adjusted to direction) [in kJ/mol]; dG [in kJ/mol];
Reaction string (adjusted to direction):
S_to_A_strain1; 1; forward; 4; 0.19137; 1 M_S_c_strain1 -> 1 M_A_c_strain1 + 1
M_X_c_strain1
A_to_B_strain1; 1; forward; -5; -3.6068; 1 M_A_c_strain1 + 1 M_X_c_strain1 -> 1
M_B_c_strain1
B_to_C_strain1; 1; forward; -5; -0.68555; 1 M_B_c_strain1 -> 1 M_X_c_strain1 + 1
M_C_c_strain1
C_to_P_strain1; 1; forward; 4; 0.19137; 1 M_X_c_strain1 + 1 M_C_c_strain1 -> 1
M_P_c_strain1
EXCHG_strain1_P_c_to_P; 1; forward; 0; 0.19137; 1 M_P_c_strain1 (<)-> 1 M_P_exchg
EXCHG_strain1_S_c_to_S; -1; reverse; 0; 0; 1 M_S_exchg (<)-> 1 M_S_c_strain1
Active reactions of exchange compartment<->environment exchange reactions:
EX_C_S_exchg; -1; reverse; NaN; NaN; (<)-> 1 M_S_exchg
EX_C_P_exchg; 1; forward; NaN; NaN; 1 M_P_exchg ->
```
