# Supplementary material for: Designing microbial communities to maximize the thermodynamic driving force for the production of chemicals
Source: PLoS Comput Biol. 2021 Jun 15;17(6):e1009093. doi: 10.1371/journal.pcbi.1009093 (PMC8232427; doi:10.1371/journal.pcbi.1009093)
Supplement: S4 Text — (PDF) [file pcbi.1009093.s004.pdf]

Designing Microbial Communities to Maximize the Thermodynamic Driving Force for the Production of Chemicals

S4 Text

Pavlos Stephanos Bekiaris[1] and Steffen Klamt[2,*]

[1]Max Planck Institute for Dynamics of Complex Technical Systems, Magdeburg, Germany

[2]Max Planck Institute for Dynamics of Complex Technical Systems, Magdeburg, Germany

* klamt@mpi-magdeburg.de

ASTHERISC report for the found community solution for kdo8p synthesis in *ecolicore2double*

The following text shows the ASTHERISC report generated for the community solution for the synthesis of 3-Deoxy-D-manno-octulosonate 8-phosphate (kdo8p) in *ecolicore2double* as discussed in the main text. The file can also be found in the ASTHERISC package's GitHub repository. [Note: The given warning for the approximation of the optimal yield does not mean that the computation failed, instead, it indicates that, with the given maximal yield approximation steps, no higher yield could be found.

```
==TARGET WITH COMMUNITY ADVANTAGE: R_EX_C_kdo8p_exchg==
WARNING: Approximated optimal yield at approximated optimal MDF for the
community could not be calculated, this is possible e.g. due to timeouts.
Optimal yield without community and with deactivated dG0=NaN reactions
without any MDF constraint [in mol/mol]: 0.74775
Optimal yield without community without any MDF constraint [in mol/mol]:
0.74775
Optimal MDF without community with minimal necessary yield as minimal yield
constraint [in kJ/mol]: 2.2737
Reached yield without community at optimal MDF solution (this yield is a
lower bound) [in mol/mol]: 0.74775
Optimal MDF with community with minimal necessary yield as minimal yield
constraint (if no warning given) [in kJ/mol]: 2.872
Approximated optimal yield with community at maximal community MDF (if no
warning given) [in mol/mol]: 0.73311
Number of active metabolites: 73

Number of active reactions | Total absolute flux per species [in
mmol/(gDW*h)]; Both with minimal absolute flux sum solution and substrate
uptake scaled to maximum:
ecoli1: 28 | 13.8547
ecoli2: 16 | 4.3598

Extra exchanges and scaled flux [in mmol/(gDW*h)] with minimal absolute flux
solution (negative flux means uptake to species, positive flux secretion from
species)
and associated metabolites with concentrations in M:
R_EXCHG_ecoli1_kdo8p_c_to_kdo8p | SCALED FLUX: 0.73311 | M_kdo8p_c_ecoli1
[3.1852e-06;0.02] | M_kdo8p_exchg [1e-06;0.0062791]
R_EXCHG_ecoli1_6pgc_c_to_6pgc | SCALED FLUX: -0.30068 | M_6pgc_c_ecoli1 [1e-
06;0.00074141] | M_6pgc_exchg [3.1852e-06;0.0023615]
R_EXCHG_ecoli1_dha_c_to_dha | SCALED FLUX: 0.60135 | M_dha_c_ecoli1
[0.00059826;0.00059905] | M_dha_exchg [0.00018783;0.00018808]
R_EXCHG_ecoli2_6pgc_c_to_6pgc | SCALED FLUX: 0.30068 | M_6pgc_c_ecoli2
[1.0145e-05;0.0075218] | M_6pgc_exchg [3.1852e-06;0.0023615]
R_EXCHG_ecoli2_dha_c_to_dha | SCALED FLUX: -0.60135 | M_dha_c_ecoli2
[5.8969e-05;5.9048e-05] | M_dha_exchg [0.00018783;0.00018808]
```

```
Metabolites with non-overlapping concentration ranges between species
[concentrations are given in M]:
M_dha_c_ecoli1 [0.00059826;0.00059905] & M_dha_c_ecoli2 [5.8969e-05;5.9048e-
05]
M_f6p_c_ecoli1 [0.00037078;0.00037128] & M_f6p_c_ecoli2 [1e-06;1.0027e-06]
M_g3p_c_ecoli1 [0.00032482;0.00032525] & M_g3p_c_ecoli2 [9.0047e-05;9.0286e-
05]
M_g6p_c_ecoli1 [0.019973;0.02] & M_g6p_c_ecoli2 [1.9629e-06;5.3238e-06]
M_13dpg_c_ecoli1 [1e-06;1e-06] & M_13dpg_c_ecoli2 [unused]
M_2ddg6p_c_ecoli1 [1e-06;0.02] & M_2ddg6p_c_ecoli2 [unused]
M_2pg_c_ecoli1 [1e-06;1.2863e-05] & M_2pg_c_ecoli2 [unused]
M_3pg_c_ecoli1 [1.9729e-05;0.00025378] & M_3pg_c_ecoli2 [unused]
M_6pgl_c_ecoli1 [unused] & M_6pgl_c_ecoli2 [1e-06;2.7122e-06]
M_amp_c_ecoli1 [1e-06;0.013333] & M_amp_c_ecoli2 [unused]
M_ara5p_c_ecoli1 [1e-06;3.8739e-06] & M_ara5p_c_ecoli2 [unused]
M_co2_c_ecoli1 [1e-06;0.001] & M_co2_c_ecoli2 [unused]
M_dhap_c_ecoli1 [unused] & M_dhap_c_ecoli2 [0.002719;0.0027262]
M_e4p_c_ecoli1 [4.1645e-06;0.0002421] & M_e4p_c_ecoli2 [unused]
M_fdp_c_ecoli1 [unused] & M_fdp_c_ecoli2 [0.0065364;0.0065712]
M_glc__D_c_ecoli1 [4.6483e-06;0.0062791] & M_glc__D_c_ecoli2 [unused]
M_glyc3p_c_ecoli1 [unused] & M_glyc3p_c_ecoli2 [1e-06;0.02]
M_kdo8p_c_ecoli1 [3.1852e-06;0.02] & M_kdo8p_c_ecoli2 [unused]
M_pep_c_ecoli1 [1e-06;1.8788e-05] & M_pep_c_ecoli2 [unused]
M_pyr_c_ecoli1 [1e-06;0.02] & M_pyr_c_ecoli2 [unused]
M_r5p_c_ecoli1 [2.2402e-05;0.0013023] & M_r5p_c_ecoli2 [unused]
M_ru5p__D_c_ecoli1 [1.1864e-06;4.5958e-06] & M_ru5p__D_c_ecoli2 [unused]
M_s7p_c_ecoli1 [6.0694e-06;0.00035284] & M_s7p_c_ecoli2 [unused]
M_xu5p__D_c_ecoli1 [1.4823e-05;0.00011302] & M_xu5p__D_c_ecoli2 [unused]
M_co2_p_ecoli1 [3.1852e-06;0.001] & M_co2_p_ecoli2 [unused]
M_glc__D_p_ecoli1 [1.4805e-05;0.02] & M_glc__D_p_ecoli2 [unused]

Bottleneck reactions (maximal driving force in order to reach OptMDF is equal
to OptMDF) of community solution:
>Bottleneck reactions (maximal driving force in order to reach OptMDF is
equal to OptMDF):
R_F6PA_ecoli1 | 1 M_f6p_c_ecoli1 (<)-> 1 M_dha_c_ecoli1 + 1 M_g3p_c_ecoli1
R_GAPD_ecoli1 | 1 M_g3p_c_ecoli1 + 1 M_nad_c_ecoli1 + 1 M_pi_c_ecoli1 (<)-> 1
M_13dpg_c_ecoli1 + 1 M_h_c_ecoli1 + 1 M_nadh_c_ecoli1
R_PGI_ecoli1 | 1 M_g6p_c_ecoli1 (<)-> 1 M_f6p_c_ecoli1
R_PIt2rpp_ecoli1 | 1 M_h_p_ecoli1 + 1 M_pi_p_ecoli1 (<)-> 1 M_h_c_ecoli1 + 1
M_pi_c_ecoli1
R_F6PA_ecoli2 | 1 M_g3p_c_ecoli2 + 1 M_dha_c_ecoli2 (<)-> 1 M_f6p_c_ecoli2
R_FBA_ecoli2 | 1 M_fdp_c_ecoli2 (<)-> 1 M_g3p_c_ecoli2 + 1 M_dhap_c_ecoli2
R_PFK_ecoli2 | 1 M_atp_c_ecoli2 + 1 M_f6p_c_ecoli2 -> 1 M_adp_c_ecoli2 + 1
M_h_c_ecoli2 + 1 M_fdp_c_ecoli2
R_TPI_ecoli2 | 1 M_dhap_c_ecoli2 (<)-> 1 M_g3p_c_ecoli2
R_EXCHG_ecoli1_dha_c_to_dha | 1 M_dha_c_ecoli1 (<)-> 1 M_dha_exchg
R_EXCHG_ecoli2_dha_c_to_dha | 1 M_dha_exchg (<)-> 1 M_dha_c_ecoli2
>Connecting metabolites:
R_F6PA_ecoli1-> R_EXCHG_ecoli1_dha_c_to_dha; M_dha_c_ecoli1
R_PGI_ecoli1-> R_F6PA_ecoli1; M_f6p_c_ecoli1
```

R_F6PA_ecoli1-> R_GAPD_ecoli1; M_g3p_c_ecoli1
R_PIt2rpp_ecoli1-> R_GAPD_ecoli1; M_pi_c_ecoli1
R_FBA_ecoli2/R_TPI_ecoli2-> R_F6PA_ecoli2; M_g3p_c_ecoli2
R_F6PA_ecoli2-> R_PFK_ecoli2; M_f6p_c_ecoli2
R_EXCHG_ecoli2_dha_c_to_dha-> R_F6PA_ecoli2; M_dha_c_ecoli2
R_PFK_ecoli2-> R_FBA_ecoli2; M_fdp_c_ecoli2
R_FBA_ecoli2-> R_TPI_ecoli2; M_dhap_c_ecoli2
R_EXCHG_ecoli1_dha_c_to_dha-> R_EXCHG_ecoli2_dha_c_to_dha; M_dha_exchg

Bottleneck reactions (driving force=OptMDF) of single-species solution as calculated back from community solution:
MDF in single species with reactions occurring in community solution [in kJ/mol]: 2.2737 (OptMDF in community was 2.872 kJ/mol, difference is 0.59831 kJ/mol)
>Bottleneck reactions (driving force=OptMDF):
R_FBA_ecoli1 | 1 M_fdp_c_ecoli1 (<)-> 1 M_dhap_c_ecoli1 + 1 M_g3p_c_ecoli1
R_GAPD_ecoli1 | 1 M_g3p_c_ecoli1 + 1 M_nad_c_ecoli1 + 1 M_pi_c_ecoli1 (<)-> 1 M_13dpg_c_ecoli1 + 1 M_h_c_ecoli1 + 1 M_nadh_c_ecoli1
R_PIt2rpp_ecoli1 | 1 M_h_p_ecoli1 + 1 M_pi_p_ecoli1 (<)-> 1 M_h_c_ecoli1 + 1 M_pi_c_ecoli1
R_TPI_ecoli1 | 1 M_dhap_c_ecoli1 (<)-> 1 M_g3p_c_ecoli1
>Connecting metabolites:
R_FBA_ecoli1-> R_TPI_ecoli1; M_dhap_c_ecoli1
R_FBA_ecoli1/R_TPI_ecoli1-> R_GAPD_ecoli1; M_g3p_c_ecoli1
R_PIt2rpp_ecoli1-> R_GAPD_ecoli1; M_pi_c_ecoli1
>Connecting metabolite concentration ranges [in M]:
~M_dhap_c_ecoli1~
In single species:
[0.0047548;0.0047574] (range 2.6175e-06)
In community:
M_dhap_c_ecoli1: [unused] (range 0)
M_dhap_c_ecoli2: [0.002719;0.0027262] (range 7.2297e-06)
~M_g3p_c_ecoli1~
In single species:
[0.00020045;0.00020045] (range 0)
In community:
M_g3p_c_ecoli1: [0.00032482;0.00032525] (range 4.3155e-07)
M_g3p_c_ecoli2: [9.0047e-05;9.0286e-05] (range 2.3943e-07)
~M_pi_c_ecoli1~
In single species:
[0.0079931;0.0079931] (range 0)
In community:
M_pi_c_ecoli1: [0.0062708;0.0062791] (range 8.3314e-06)
M_pi_c_ecoli2: [3.6645e-05;0.0062791] (range 0.0062425)

Detailed reaction-wise solution reports:
~Detailed solution of community solution~
Active reactions associated with species ecoli1 (Reaction ID; Flux [in mmol/(gDW*h)]; Direction; dG0 (adjusted to direction) [in kJ/mol]; dG [in kJ/mol]; Reaction string (adjusted to direction):

A5PISO_ecoli1; 0.73311; forward; -2.4481; -2.872; 1 M_ru5p__D_c_ecoli1 (<)-> 1 M_ara5p_c_ecoli1

ADK1_ecoli1; 0.16554; forward; -0.19453; -4.636; 1 M_amp_c_ecoli1 + 1 M_atp_c_ecoli1 (<)-> 2 M_adp_c_ecoli1

ATPS4rpp_ecoli1; 0.76351; forward; -33.8921; -18.5988; 1 M_adp_c_ecoli1 + 1 M_pi_c_ecoli1 + 4 M_h_p_ecoli1 (<)-> 1 M_atp_c_ecoli1 + 3 M_h_c_ecoli1 + 1 M_h2o_c_ecoli1

CO2tpp_ecoli1; -0.13514; reverse; NaN; NaN; 1 M_co2_c_ecoli1 (<)-> 1 M_co2_p_ecoli1

CYTBO3_4pp_ecoli1; 0.7027; forward; -55.284; -50.4354; 4 M_h_c_ecoli1 + 0.5 M_o2_c_ecoli1 + 1 M_q8h2_c_ecoli1 -> 1 M_h2o_c_ecoli1 + 1 M_q8_c_ecoli1 + 4 M_h_p_ecoli1

EDA_ecoli1; 0.16554; forward; 16.2402; -13.0839; 1 M_2ddg6p_c_ecoli1 -> 1 M_g3p_c_ecoli1 + 1 M_pyr_c_ecoli1

EDD_ecoli1; 0.16554; forward; -42.8448; -24.0099; 1 M_6pgc_c_ecoli1 -> 1 M_2ddg6p_c_ecoli1 + 1 M_h2o_c_ecoli1

ENO_ecoli1; 0.56757; forward; -3.8109; -9.413; 1 M_2pg_c_ecoli1 (<)-> 1 M_h2o_c_ecoli1 + 1 M_pep_c_ecoli1

F6PA_ecoli1; 0.60135; forward; 15.8529; -2.872; 1 M_f6p_c_ecoli1 (<)-> 1 M_dha_c_ecoli1 + 1 M_g3p_c_ecoli1

GAPD_ecoli1; 0.56757; forward; 4.6027; -2.872; 1 M_g3p_c_ecoli1 + 1 M_nad_c_ecoli1 + 1 M_pi_c_ecoli1 (<)-> 1 M_13dpg_c_ecoli1 + 1 M_h_c_ecoli1 + 1 M_nadh_c_ecoli1

GLCt2pp_ecoli1; 1; forward; 0; -2.872; 1 M_glc__D_p_ecoli1 + 1 M_h_p_ecoli1 -> 1 M_glc__D_c_ecoli1 + 1 M_h_c_ecoli1

GND_ecoli1; 0.13514; forward; 10.332; -26.4811; 1 M_6pgc_c_ecoli1 + 1 M_nadp_c_ecoli1 -> 1 M_co2_c_ecoli1 + 1 M_nadph_c_ecoli1 + 1 M_ru5p__D_c_ecoli1

H2Otpp_ecoli1; -1.3007; reverse; NaN; NaN; 1 M_h2o_c_ecoli1 (<)-> 1 M_h2o_p_ecoli1

HEX1_ecoli1; 1; forward; -17.901; -17.7523; 1 M_atp_c_ecoli1 + 1 M_glc__D_c_ecoli1 -> 1 M_adp_c_ecoli1 + 1 M_g6p_c_ecoli1 + 1 M_h_c_ecoli1

KDOPS_ecoli1; 0.73311; forward; -66.5142; -21.015; 1 M_ara5p_c_ecoli1 + 1 M_h2o_c_ecoli1 + 1 M_pep_c_ecoli1 -> 1 M_kdo8p_c_ecoli1 + 1 M_pi_c_ecoli1

NADH16pp_ecoli1; 0.7027; forward; -81.2018; -75.4941; 4 M_h_c_ecoli1 + 1 M_nadh_c_ecoli1 + 1 M_q8_c_ecoli1 (<)-> 1 M_nad_c_ecoli1 + 1 M_q8h2_c_ecoli1 + 3 M_h_p_ecoli1

NADTRHD_ecoli1; 0.13514; forward; 0.13056; -8.3004; 1 M_nad_c_ecoli1 + 1 M_nadph_c_ecoli1 -> 1 M_nadh_c_ecoli1 + 1 M_nadp_c_ecoli1

O2tpp_ecoli1; 0.35135; forward; NaN; NaN; 1 M_o2_p_ecoli1 (<)-> 1 M_o2_c_ecoli1

PGI_ecoli1; 1; forward; 7.0102; -2.872; 1 M_g6p_c_ecoli1 (<)-> 1 M_f6p_c_ecoli1

PGK_ecoli1; -0.56757; reverse; 19.3156; -2.872; 1 M_13dpg_c_ecoli1 + 1 M_adp_c_ecoli1 (<)-> 1 M_3pg_c_ecoli1 + 1 M_atp_c_ecoli1

PGM_ecoli1; -0.56757; reverse; -4.5204; -2.872; 1 M_3pg_c_ecoli1 (<)-> 1 M_2pg_c_ecoli1

PIt2rpp_ecoli1; 0.43243; forward; 0; -2.872; 1 M_h_p_ecoli1 + 1 M_pi_p_ecoli1 (<)-> 1 M_h_c_ecoli1 + 1 M_pi_c_ecoli1

PPS_ecoli1; 0.16554; forward; 0.41266; -27.5706; 1 M_atp_c_ecoli1 + 1 M_h2o_c_ecoli1 + 1 M_pyr_c_ecoli1 -> 1 M_amp_c_ecoli1 + 2 M_h_c_ecoli1 + 1 M_pep_c_ecoli1 + 1 M_pi_c_ecoli1
RPE_ecoli1; -0.39865; reverse; -3.388; -2.872; 1 M_xu5p__D_c_ecoli1 (<)-> 1 M_ru5p__D_c_ecoli1
RPI_ecoli1; 0.19932; forward; 4.4117; -2.872; 1 M_r5p_c_ecoli1 (<)-> 1 M_ru5p__D_c_ecoli1
TALA_ecoli1; -0.19932; reverse; 3.4773; -2.872; 1 M_e4p_c_ecoli1 + 1 M_f6p_c_ecoli1 (<)-> 1 M_g3p_c_ecoli1 + 1 M_s7p_c_ecoli1
TKT1_ecoli1; -0.19932; reverse; -1.5468; -2.872; 1 M_g3p_c_ecoli1 + 1 M_s7p_c_ecoli1 (<)-> 1 M_r5p_c_ecoli1 + 1 M_xu5p__D_c_ecoli1
TKT2_ecoli1; -0.19932; reverse; -5.8438; -12.9378; 1 M_f6p_c_ecoli1 + 1 M_g3p_c_ecoli1 (<)-> 1 M_e4p_c_ecoli1 + 1 M_xu5p__D_c_ecoli1
EXCHG_ecoli1_h_p_to_h; 0.43243; forward; NaN; NaN; 1 M_h_p_ecoli1 (<)-> 1 M_h_exchg
EXCHG_ecoli1_co2_p_to_co2; 0.13514; forward; 0; -17.1231; 1 M_co2_p_ecoli1 (<)-> 1 M_co2_exchg
EXCHG_ecoli1_kdo8p_c_to_kdo8p; 0.73311; forward; 0; -24.549; 1 M_kdo8p_c_ecoli1 -> 1 M_kdo8p_exchg
EXCHG_ecoli1_glc__D_p_to_glc__D; -1; reverse; 0; -15.4049; 1 M_glc__D_exchg (<)-> 1 M_glc__D_p_ecoli1
EXCHG_ecoli1_h2o_p_to_h2o; 1.3007; forward; NaN; NaN; 1 M_h2o_p_ecoli1 (<)-> 1 M_h2o_exchg
EXCHG_ecoli1_6pgc_c_to_6pgc; -0.30068; reverse; 0; -2.872; 1 M_6pgc_exchg -> 1 M_6pgc_c_ecoli1
EXCHG_ecoli1_pi_p_to_pi; -0.43243; reverse; 0; -2.872; 1 M_pi_exchg (<)-> 1 M_pi_p_ecoli1
EXCHG_ecoli1_o2_p_to_o2; -0.35135; reverse; 0; -27.421; 1 M_o2_exchg (<)-> 1 M_o2_p_ecoli1
EXCHG_ecoli1_dha_c_to_dha; 0.60135; forward; 0; -2.872; 1 M_dha_c_ecoli1 -> 1 M_dha_exchg
Active reactions associated with species ecoli2 (Reaction ID; Flux [in mmol/(gDW*h)]; Direction; dG0 (adjusted to direction) [in kJ/mol]; dG [in kJ/mol]; Reaction string (adjusted to direction):
ATPS4rpp_ecoli2; 0.30068; forward; -33.8921; -2.872; 1 M_adp_c_ecoli2 + 1 M_pi_c_ecoli2 + 4 M_h_p_ecoli2 (<)-> 1 M_atp_c_ecoli2 + 1 M_h2o_c_ecoli2 + 3 M_h_c_ecoli2
CYTBO3_4pp_ecoli2; 0.30068; forward; -55.284; -50.4354; 4 M_h_c_ecoli2 + 0.5 M_o2_c_ecoli2 + 1 M_q8h2_c_ecoli2 -> 1 M_h2o_c_ecoli2 + 4 M_h_p_ecoli2 + 1 M_q8_c_ecoli2
F6PA_ecoli2; -0.60135; reverse; 15.8529; -2.872; 1 M_g3p_c_ecoli2 + 1 M_dha_c_ecoli2 (<)-> 1 M_f6p_c_ecoli2
FBA_ecoli2; 0.30068; forward; 22.3932; -2.872; 1 M_fdp_c_ecoli2 (<)-> 1 M_g3p_c_ecoli2 + 1 M_dhap_c_ecoli2
G3PD2_ecoli2; -0.10023; reverse; 25.6158; -47.9447; 1 M_h_c_ecoli2 + 1 M_nadph_c_ecoli2 + 1 M_dhap_c_ecoli2 (<)-> 1 M_nadp_c_ecoli2 + 1 M_glyc3p_c_ecoli2
G3PD5_ecoli2; 0.10023; forward; -109.0556; -89.4499; 1 M_glyc3p_c_ecoli2 + 1 M_q8_c_ecoli2 -> 1 M_q8h2_c_ecoli2 + 1 M_dhap_c_ecoli2
G6PDH2r_ecoli2; 0.30068; forward; -3.9232; -2.872; 1 M_nadp_c_ecoli2 + 1 M_g6p_c_ecoli2 (<)-> 1 M_h_c_ecoli2 + 1 M_nadph_c_ecoli2 + 1 M_6pgl_c_ecoli2

H2Otpp_ecoli2; -0.30068; reverse; NaN; NaN; 1 M_h2o_c_ecoli2 (<)-> 1 M_h2o_p_ecoli2
NADH16pp_ecoli2; 0.20045; forward; -81.2018; -75.4941; 4 M_h_c_ecoli2 + 1 M_nadh_c_ecoli2 + 1 M_q8_c_ecoli2 (<)-> 1 M_nad_c_ecoli2 + 3 M_h_p_ecoli2 + 1 M_q8h2_c_ecoli2
NADTRHD_ecoli2; 0.20045; forward; 0.13056; -8.3004; 1 M_nad_c_ecoli2 + 1 M_nadph_c_ecoli2 -> 1 M_nadp_c_ecoli2 + 1 M_nadh_c_ecoli2
O2tpp_ecoli2; 0.15034; forward; NaN; NaN; 1 M_o2_p_ecoli2 (<)-> 1 M_o2_c_ecoli2
PFK_ecoli2; 0.30068; forward; -18.9474; -2.872; 1 M_atp_c_ecoli2 + 1 M_f6p_c_ecoli2 -> 1 M_adp_c_ecoli2 + 1 M_h_c_ecoli2 + 1 M_fdp_c_ecoli2
PGI_ecoli2; -0.30068; reverse; 7.0102; -2.872; 1 M_f6p_c_ecoli2 (<)-> 1 M_g6p_c_ecoli2
PGL_ecoli2; 0.30068; forward; -22.5233; -5.3381; 1 M_h2o_c_ecoli2 + 1 M_6pgl_c_ecoli2 -> 1 M_h_c_ecoli2 + 1 M_6pgc_c_ecoli2
PIt2rpp_ecoli2; 0.30068; forward; 0; -2.872; 1 M_h_p_ecoli2 + 1 M_pi_p_ecoli2 (<)-> 1 M_h_c_ecoli2 + 1 M_pi_c_ecoli2
TPI_ecoli2; 0.30068; forward; 5.5753; -2.872; 1 M_dhap_c_ecoli2 (<)-> 1 M_g3p_c_ecoli2
EXCHG_ecoli2_h_p_to_h; 0.30068; forward; NaN; NaN; 1 M_h_p_ecoli2 (<)-> 1 M_h_exchg
EXCHG_ecoli2_h2o_p_to_h2o; 0.30068; forward; NaN; NaN; 1 M_h2o_p_ecoli2 (<)-> 1 M_h2o_exchg
EXCHG_ecoli2_6pgc_c_to_6pgc; 0.30068; forward; 0; -11.0652; 1 M_6pgc_c_ecoli2 -> 1 M_6pgc_exchg
EXCHG_ecoli2_pi_p_to_pi; -0.30068; reverse; 0; -15.6143; 1 M_pi_exchg (<)-> 1 M_pi_p_ecoli2
EXCHG_ecoli2_o2_p_to_o2; -0.15034; reverse; 0; -27.421; 1 M_o2_exchg (<)-> 1 M_o2_p_ecoli2
EXCHG_ecoli2_dha_c_to_dha; -0.60135; reverse; 0; -2.872; 1 M_dha_exchg -> 1 M_dha_c_ecoli2
Active reactions of exchange compartment<->environment exchange reactions:
EX_C_kdo8p_exchg; 0.73311; forward; NaN; NaN; 1 M_kdo8p_exchg ->
EX_C_h_exchg; 0.73311; forward; NaN; NaN; 1 M_h_exchg (<)->
EX_C_co2_exchg; 0.13514; forward; NaN; NaN; 1 M_co2_exchg (<)->
EX_C_glc__D_exchg; -1; reverse; NaN; NaN; (<)-> 1 M_glc__D_exchg
EX_C_h2o_exchg; 1.6014; forward; NaN; NaN; 1 M_h2o_exchg (<)->
EX_C_o2_exchg; -0.50169; reverse; NaN; NaN; (<)-> 1 M_o2_exchg
EX_C_pi_exchg; -0.73311; reverse; NaN; NaN; (<)-> 1 M_pi_exchg

~Detailed solution of single-strain solution (recalculated back from community solution reactions)~
Active reactions associated with species ecoli1 (Reaction ID; Flux [in mmol/(gDW*h)]; Direction; dG0 (adjusted to direction) [in kJ/mol]; dG [in kJ/mol]; Reaction string (adjusted to direction):
A5PISO_ecoli1; 0.74775; forward; -2.4481; -2.2737; 1 M_ru5p__D_c_ecoli1 (<)-> 1 M_ara5p_c_ecoli1
ATPS4rpp_ecoli1; 0.74775; forward; -33.8921; -19.1979; 1 M_adp_c_ecoli1 + 1 M_pi_c_ecoli1 + 4 M_h_p_ecoli1 (<)-> 1 M_atp_c_ecoli1 + 3 M_h_c_ecoli1 + 1 M_h2o_c_ecoli1

CO2tpp_ecoli1; -0.018018; reverse; NaN; NaN; 1 M_co2_c_ecoli1 (<)-> 1 M_co2_p_ecoli1

CYTBO3_4pp_ecoli1; 0.78378; forward; -55.284; -74.9844; 4 M_h_c_ecoli1 + 0.5 M_o2_c_ecoli1 + 1 M_q8h2_c_ecoli1 -> 1 M_h2o_c_ecoli1 + 1 M_q8_c_ecoli1 + 4 M_h_p_ecoli1

ENO_ecoli1; 0.74775; forward; -3.8109; -2.2737; 1 M_2pg_c_ecoli1 (<)-> 1 M_h2o_c_ecoli1 + 1 M_pep_c_ecoli1

FBA_ecoli1; 0.4955; forward; 22.3932; -2.2737; 1 M_fdp_c_ecoli1 (<)-> 1 M_dhap_c_ecoli1 + 1 M_g3p_c_ecoli1

G6PDH2r_ecoli1; 0.018018; forward; -3.9232; -2.2737; 1 M_g6p_c_ecoli1 + 1 M_nadp_c_ecoli1 (<)-> 1 M_6pgl_c_ecoli1 + 1 M_h_c_ecoli1 + 1 M_nadph_c_ecoli1

GAPD_ecoli1; 0.74775; forward; 4.6027; -2.2737; 1 M_g3p_c_ecoli1 + 1 M_nad_c_ecoli1 + 1 M_pi_c_ecoli1 (<)-> 1 M_13dpg_c_ecoli1 + 1 M_h_c_ecoli1 + 1 M_nadh_c_ecoli1

GLCt2pp_ecoli1; 1; forward; 0; -9.2065; 1 M_glc__D_p_ecoli1 + 1 M_h_p_ecoli1 -> 1 M_glc__D_c_ecoli1 + 1 M_h_c_ecoli1

GND_ecoli1; 0.018018; forward; 10.332; -45.7398; 1 M_6pgc_c_ecoli1 + 1 M_nadp_c_ecoli1 -> 1 M_co2_c_ecoli1 + 1 M_nadph_c_ecoli1 + 1 M_ru5p__D_c_ecoli1

H2Otpp_ecoli1; -1.5135; reverse; NaN; NaN; 1 M_h2o_c_ecoli1 (<)-> 1 M_h2o_p_ecoli1

HEX1_ecoli1; 1; forward; -17.901; -2.2737; 1 M_atp_c_ecoli1 + 1 M_glc__D_c_ecoli1 -> 1 M_adp_c_ecoli1 + 1 M_g6p_c_ecoli1 + 1 M_h_c_ecoli1

KDOPS_ecoli1; 0.74775; forward; -66.5142; -28.9261; 1 M_ara5p_c_ecoli1 + 1 M_h2o_c_ecoli1 + 1 M_pep_c_ecoli1 -> 1 M_kdo8p_c_ecoli1 + 1 M_pi_c_ecoli1

NADH16pp_ecoli1; 0.78378; forward; -81.2018; -50.9452; 4 M_h_c_ecoli1 + 1 M_nadh_c_ecoli1 + 1 M_q8_c_ecoli1 (<)-> 1 M_nad_c_ecoli1 + 1 M_q8h2_c_ecoli1 + 3 M_h_p_ecoli1

NADTRHD_ecoli1; 0.036036; forward; 0.13056; -8.3004; 1 M_nad_c_ecoli1 + 1 M_nadph_c_ecoli1 -> 1 M_nadh_c_ecoli1 + 1 M_nadp_c_ecoli1

O2tpp_ecoli1; 0.39189; forward; NaN; NaN; 1 M_o2_p_ecoli1 (<)-> 1 M_o2_c_ecoli1

PFK_ecoli1; 0.4955; forward; -18.9474; -12.3868; 1 M_atp_c_ecoli1 + 1 M_f6p_c_ecoli1 -> 1 M_adp_c_ecoli1 + 1 M_fdp_c_ecoli1 + 1 M_h_c_ecoli1

PGI_ecoli1; 0.98198; forward; 7.0102; -2.2737; 1 M_g6p_c_ecoli1 (<)-> 1 M_f6p_c_ecoli1

PGK_ecoli1; -0.74775; reverse; 19.3156; -2.2737; 1 M_13dpg_c_ecoli1 + 1 M_adp_c_ecoli1 (<)-> 1 M_3pg_c_ecoli1 + 1 M_atp_c_ecoli1

PGL_ecoli1; 0.018018; forward; -22.5233; -21.4495; 1 M_6pgl_c_ecoli1 + 1 M_h2o_c_ecoli1 -> 1 M_6pgc_c_ecoli1 + 1 M_h_c_ecoli1

PGM_ecoli1; -0.74775; reverse; -4.5204; -2.2737; 1 M_3pg_c_ecoli1 (<)-> 1 M_2pg_c_ecoli1

PIt2rpp_ecoli1; 0.74775; forward; 0; -2.2737; 1 M_h_p_ecoli1 + 1 M_pi_p_ecoli1 (<)-> 1 M_h_c_ecoli1 + 1 M_pi_c_ecoli1

RPE_ecoli1; -0.48649; reverse; -3.388; -2.2737; 1 M_xu5p__D_c_ecoli1 (<)-> 1 M_ru5p__D_c_ecoli1

RPI_ecoli1; 0.24324; forward; 4.4117; -17.2002; 1 M_r5p_c_ecoli1 (<)-> 1 M_ru5p__D_c_ecoli1

TALA_ecoli1; -0.24324; reverse; 3.4773; -2.2737; 1 M_e4p_c_ecoli1 + 1 M_f6p_c_ecoli1 (<)-> 1 M_g3p_c_ecoli1 + 1 M_s7p_c_ecoli1

TKT1_ecoli1; -0.24324; reverse; -1.5468; -2.2737; 1 M_g3p_c_ecoli1 + 1 M_s7p_c_ecoli1 (<)-> 1 M_r5p_c_ecoli1 + 1 M_xu5p__D_c_ecoli1
TKT2_ecoli1; -0.24324; reverse; -5.8438; -2.2737; 1 M_f6p_c_ecoli1 + 1 M_g3p_c_ecoli1 (<)-> 1 M_e4p_c_ecoli1 + 1 M_xu5p__D_c_ecoli1
TPI_ecoli1; 0.4955; forward; 5.5753; -2.2737; 1 M_dhap_c_ecoli1 (<)-> 1 M_g3p_c_ecoli1
EXCHG_ecoli1_h_p_to_h; 0.74775; forward; NaN; NaN; 1 M_h_p_ecoli1 (<)-> 1 M_h_exchg
EXCHG_ecoli1_co2_p_to_co2; 0.018018; forward; 0; -17.1231; 1 M_co2_p_ecoli1 (<)-> 1 M_co2_exchg
EXCHG_ecoli1_kdo8p_c_to_kdo8p; 0.74775; forward; 0; -24.549; 1 M_kdo8p_c_ecoli1 -> 1 M_kdo8p_exchg
EXCHG_ecoli1_glc__D_p_to_glc__D; -1; reverse; 0; -24.549; 1 M_glc__D_exchg (<)-> 1 M_glc__D_p_ecoli1
EXCHG_ecoli1_h2o_p_to_h2o; 1.5135; forward; NaN; NaN; 1 M_h2o_p_ecoli1 (<)-> 1 M_h2o_exchg
EXCHG_ecoli1_pi_p_to_pi; -0.74775; reverse; 0; -15.4049; 1 M_pi_exchg (<)-> 1 M_pi_p_ecoli1
EXCHG_ecoli1_o2_p_to_o2; -0.39189; reverse; 0; -39.9538; 1 M_o2_exchg (<)-> 1 M_o2_p_ecoli1
Active reactions of exchange compartment<->environment exchange reactions:
EX_C_kdo8p_exchg; 0.74775; forward; NaN; NaN; 1 M_kdo8p_exchg ->
EX_C_h_exchg; 0.74775; forward; NaN; NaN; 1 M_h_exchg (<)->
EX_C_co2_exchg; 0.018018; forward; NaN; NaN; 1 M_co2_exchg (<)->
EX_C_glc__D_exchg; -1; reverse; NaN; NaN; (<)-> 1 M_glc__D_exchg
EX_C_h2o_exchg; 1.5135; forward; NaN; NaN; 1 M_h2o_exchg (<)->
EX_C_o2_exchg; -0.39189; reverse; NaN; NaN; (<)-> 1 M_o2_exchg
EX_C_pi_exchg; -0.74775; reverse; NaN; NaN; (<)-> 1 M_pi_exchg
