# Supplementary material for: Designing microbial communities to maximize the thermodynamic driving force for the production of chemicals
Source: PLoS Comput Biol. 2021 Jun 15;17(6):e1009093. doi: 10.1371/journal.pcbi.1009093 (PMC8232427; doi:10.1371/journal.pcbi.1009093)
Supplement: Attachment — Submitted filename: Response_Letter_final.docx [file pcbi.1009093.s006.docx]

Response to the comments of the reviewers

**Manuscript:**

**“Designing Microbial Communities to Maximize the Thermodynamic Driving Force for the Production of Chemicals”**

**by Pavlos Stephanos Bekiaris and Steffen Klamt**

We thank all reviewers for the careful evaluation of our manuscript and the constructive comments. Below we give a detailed response to all suggestions and questions of the reviewers and describe the respective changes in the manuscript (changes are also highlighted in the revised manuscript file).

**Reviewer #1**

The authors present a new method, ASTHERISC, for designing synthetic microbial communities with optimal thermodynamic driving force for the production of a target compound. The method is novel with regard to being the first community design method that accounts for thermodynamics, and I believe it is quite relevant to other people working in this field. However, I think the authors should discuss some limitations in a bit more detail. Please see my comments bellow.

We thank the reviewer for the positive general evaluation of the manuscript. Below, we provide a detailed answer to the remaining issues.

- The theoretical model of division of labor (DoL) assumes that there is no cost associated with the transport of the exchanged metabolic intermediates. For instance, in Figure 1, if one adds a cost term for the transport of M, the DoL model actually has an increased metabolic burden. This issue is only very briefly mentioned in the discussion, but given that it can make the whole concept unfeasible in a real scenario, I think it should be better discussed. For instance, the authors could have tested the sensitivity of the solutions with respect to the addition of an exchange cost (not just a limit on the total number of exchanges).

We agree with the reviewer that the introduction of transport costs may change the results (for both “classical DoL” as in Fig. 1 as well as for our ASTHERISC approach in Fig. 2) and that this should be discussed. First of all, as mentioned in the Supplementary Text S2, in all calculations we demanded thermodynamic feasibility of the exchange reactions between the organisms and the exchange compartment, namely by setting dG’0=0 kJ/mol for these transport reactions. This already implies that there must be a gradient between the metabolite concentrations in the two compartments and that the metabolite can only flow from higher to lower concentrations. Even though this does not directly account for energetic costs, it ensures thermodynamic feasibility and already limits the number of possible solutions.

To analyze the effects of additional transport costs (e.g. via antiport mechanisms with protons) we added to each introduced non-default (extra) exchange reaction in *ecolicore2double* transport costs of 1/3 ATP (with a stoichiometrically balanced usage of 1/3 H2O and production of 1/3 ADP and 1/3 Pi). We then re-run all calculations with this modified *ecolicore2double* network (for all four yield thresholds and with (a) an infinite and (b) 9 allowed extra exchanges as before). We found that the percentage of products, where ASTHERISC finds a community with an MDF advantage, does not change dramatically, indeed, it could, in some scenarios, even increase compared to the case without ATP transport costs. This somewhat surprising result can be reasoned with the fact that now also the single strain must produce some ATP to export the (end) product which may change the structure of the active network in some cases. Nevertheless, we also found products where the network without transport costs brings an MDF advantage in a community where the network with transport costs does not. Hence, these results suggest that the introduction of (ATP) transport costs may change the specific results returned by ASTHERISC, but it does not affect the general feasibility of finding communities with enhanced thermodynamic driving forces. We also added a short note on this in the Discussion section.

- Also, the authors add artificial exchange reactions to transport the metabolites between the cytosol and the growth medium, which leads to the exchange of metabolites, like phosphorylated sugars, that in principle cannot be transported outside the cell (glyc3p and f6p are reported to be among the most frequently exchanged). Why not simply use the transporters that are already present in the models ?

First of all, we would like to mention that there are some phosphorylated metabolites, such as glucose-6-phosphat, that can be transported (e.g. from the environment into the cytosol in E. coli). However, we agree with the reviewer that many of the set exchangeable metabolites, especially phosphorylated species, might not be exchangeable *in vivo*. We nevertheless included these transporters since in our study we wanted to show the full theoretical and combinatorial potential of community interactions (similar as was done in the DOLMN paper; Thomas et al., 2019, Designing metabolic division of labor in microbial communities, *mSystems, 2019, 4, no. 2*) without limiting it too much with respect to known transporters (or transporters contained in the model). Clearly, once a community solution is selected for implementation, this aspect has to be taken into account (and can be enforced by the algorithm).

- Another aspect which is not discussed is what would happen during the scale-up to very large (i.e. not well-mixed bioreactors). Could the rate of diffusion of the metabolic intermediates become a bottleneck?

The reviewer is right that diffusion might be a problem in bioreactors. But this is actually a relevant issue for any division-of-labor (or division-of-pathway) strategy or even for any (also natural) communities used in a bioprocess and is thus not a specific issue for the design principle of ASTHERISC. In reality, possible solutions would include the construction of suitable bioreactors or the use of high-density cell cultures in the production phase. However, a full discussion of those technical issues goes beyond the scope of the paper.

- The results presented in Table 1 show the fraction of cases where the optimal MDF is increased. But it is not mentioned how marginal this increase can be. I think the application of ASTHERISC is mainly relevant to the case illustrated in Fig 2, where a pathway with negative OptMDF can be split between species to obtain a positive MDF. How many of the cases presented in table 1 are actually of this kind?

First of all, we would like to point out that we demanded a minimum MDF gain of +0.2 kJ/mol (as described in the section “Applying ASTERISC to multi-strain community models of E. coli”; we now also added a note to the table legend). Hence, all results shown in Table 1 imply a significant gain in thermodynamic driving force and we believe that those cases are also relevant and interesting. However, we agree with the reviewer that an MDF sign change is the most significant possible advantage of a community designed with ASTHERISC. We checked in how many solutions this was the case and found that a sign change in the MDF could be observed for 7 metabolites in all runs with a yield threshold of 98% with ecolicore2double and ecolicore2triple, and 3 metabolites in all runs with iML1515double. Hence, in our simulations, there are relatively few MDF sign changes, which is likely also a consequence of the very broad metabolite concentration ranges allowed in the model (but this may change when choosing narrower concentration ranges). We inserted this information in the Results section (below Table 1).

- Why are the metabolite concentrations specified in M (i.e. mol/L)? What is the volume referring to in this case? Is it the cell volume? Shouldn't these values be 1000-fold lower, i.e., in the mM range ?

We used unit M in all calculations since OptMDFpathway is working with this unit. In the manuscript we chose mM only to improve readability (e.g., in Table 4). The volume is, as usual, referring to the cell volume. In the example in Figure 2 we indeed chose high concentrations (1-10 M) but this is only for illustration and works as well with 1 – 10mM. For the calculations with the E. coli models we chose a standard concentration range of 1 µM to 20 mM for internal metabolites and 1 µM to 10 M for external metabolites (see Supplementary Text S2), which is also typical for those calculations. Generally, we would like to point out that the chosen unit (M or mM) does not have an impact on the calculations (as long as they are correctly interpreted).

- I don't understand why the authors opted to block the reactions with unknown dG0's, which is essentially the same as removing them from the models. I think it would be more realistic, and biologically meaningful, to leave them unconstrained, since in reality these reactions would be able to carry flux, potentially deviating flux from the target pathway.

Indeed, as proposed by the reviewer, in the first ASTHERISC runs we allowed reactions with unknown dG’0 to be used by assuming a free (infinitely negative) dG’0. As a result, ASTHERISC returned many solutions, which often used the reactions with the free dG’0 values with partially very unfavorable (and unlikely) metabolite concentrations (e.g., very low substrate concentrations and very high product concentrations for those reactions). This implies that these reactions would have extremely low (negative) dG’0 values to run in reality. Hence, in these cases, the network gets thermodynamic driving force for free. In fact, flux distributions may arise, that are thermodynamically infeasible (e.g., including a cycle) just because the dG’0 of one participating reaction is not specified. Since this manuscript aims to present the general idea of thermodynamics-based community design and because, fortunately, the reactions without dG’0 were generally not part of central metabolic pathways, we decided to block these reactions in order to get consistent results, albeit in a reduced network. We also tested a simplified and naive way of giving all the relevant reactions a dG’0 of 0 kJ/mol, but, again, several returned ASTHERISC solutions relied on those reactions and appeared unrealistic or even thermodynamically infeasible.

We added a brief explanation on our handling with reactions with a free dG’0 in the Methods section: “Reactions for which no dG’0 could be found or calculated (93 reactions (18%) in EColiCore2 and 646 (24%) in iML1515), were blocked in the model (rate fixed to zero) to avoid the calculation of thermodynamically infeasible solutions when leaving the dG’0 unconstrained.”

- I didn't see any mention about uncertainty in the dG0 calculations, which is one of the outputs returned by eQuilibrator. Were the uncertainties ignored? Considering that some can be quite large, shouldn't they be included in equation 9 (i.e. by replacing the equality with lower/upper bounds)?

Although our method is, in principle, able to include dG’0 uncertainties in the calculations, we decided to not do so, since inclusion of uncertainties may, for example, again enforce thermodynamically infeasible cycles in the given solutions (think of a cycle where the sum of all dG’0 should be zero and where you now reduce the dG’0 of all involved reactions to its lower bounds – the sum of the dG’0 will then become negative). We added a brief explanation of our handling with the uncertainties in the Text S2.

- The algorithm doesn't actually propose any strain design targets to implement the mutant strains. It is only briefly mentioned during the discussion that this is something that must be done after computing the optimal pathway. However, considering that the title of the paper is Designing Microbial Communities (...), I think the authors should elaborate a bit more
on this aspect.

We agree with the reviewer that it needs to be mentioned how the found community solutions translate to the respective strain designs. We extended the Discussion in the manuscript accordingly:

“First of all, designing those strains will require the deactivation of certain metabolic reactions (e.g., through gene knockouts) according to the found community solution. Finding suitable knockout strategies that leave only the relevant pathway segments active in the respective strains can be computed by dedicated strain design algorithms, e.g. based on minimal cut sets. Implementing the respective gene knockout strategies may lead to unstable strains or even prevent growth. While the (stoichiometric) feasibility of growth may be demanded when computing the respective intervention strategies, it might be more practical to use two-stage processes, where the strains can first grow before a pure production phase is initiated (e.g. via dynamic metabolic switches). “

- Small suggestion: all the four tables presented could be more intuitively represented as plots.

We thank the reviewer for this suggestion, and we indeed tried to construct those plots. However, especially for Tables 2 and 3, where we have 3 values per network per yield, this becomes really cluttered and is then, in our opinion, not of much value.

**Reviewer #2**

The comments are also attached as a pdf. It might be easier to read the equations there.

\### Peer review of *Designing Microbial Communities to Maximize the Thermodynamic Driving Force for the Production of Chemicals*

The authors present an interesting algorithm for the computational design of single-species communities that maximise the thermodynamic driving force of producing a certain target metabolite. The paper is written in a clear and educational style, which makes it pleasant to read. The algorithm is potentially important, and has novelty for both the modelling of microbial communities and the design of bio-based production. Although I encountered some issues that I think should be resolved, I think the manuscript is fit for publication in Plos Computational Biology after some major revisions.

My main criticism is related to the strong and non-negligible assumptions that the ASTHERISC algorithm is based on. I understand that making these assumptions is necessary: the modelling of microbial communities is difficult, and some assumptions are needed to make computational problems feasible. However, I think the reader should be guided much more in interpreting the consequences of these assumptions. My remarks, ordered per section, can be found below.

We thank the reviewer for the generally positive view on our manuscript. Regarding the raised issues, we provide detailed answers below.

*#### Remarks Introduction* ##### The example described in Figure 1
Although I appreciate the attempt to introduce the concept of DoL in a very basic way, the example model is a bit too basic for my taste. At a first glance, I am left with the idea that the assumptions that are made are necessary to draw the conclusions that the authors want to make. I think this is not the impression that the authors want to leave, because their argument is much more general. Also, the statement “Hence, it is unlikely that a DoL strategy will be advantageous with respect to the metabolic burden …” is repeated a couple of times in the introduction, and I think it is not entirely correct. See my explanation below on how this could be nuanced. The two assumptions that I find too specific are:
- the saturation functions only depend on the substrate of the reaction, rather than also on the product.

- the same amount of enzymes E1 and E2 is needed for a specific flux

I know that the authors already state that the second assumption does not affect the conclusions, but the reader is still left wondering what the effect of this assumption might be.
I would suggest two edits:

1. I think it is fairly straightforward to write a small mathematical proof that DoL does not give an advantage when the metabolite concentrations are unchanged, i.e., the saturation functions remain the same. This proof can be added to the SI, and can be referred to in this Introduction. I have added an outline for the proof below.
Adding this proof would also allow for a more exact statement. The authors now state: *“Hence, it is unlikely that a DoL strategy will be advantageous with respect to the metabolic burden … ”* But this should actually be something like: *“Hence, it is unlikely that a DoL strategy will be advantageous with respect to the metabolic burden, as long as the kinetic advantage of having different metabolite concentrations in the different strains do not outweigh the added costs of metabolite transport.”* The statement is a bit less strong, but definitely more correct. Also, adjusting this statement would allow the authors to give a preview on the rest of their work, because adding a growth advantage by having different metabolite concentrations is exactly what they are going to do, albeit focusing on thermodynamics and not on kinetics.
2. In this Introduction I would at least remove the first assumption, and just make the saturation functions depend on all metabolite concentrations. This does not change the reasoning at all. Personally, I would also skip the second assumption, but I realize that this is a matter of taste. Generalising this here might complicate matters. It is also not really necessary as long as you can refer to the general proof in the SI.

An idea for a proof <Here it follows a sketch of the proof given by the reviewer>

We are very grateful for this excellent suggestion and we totally agree with the advice given by the reviewer. Moreover, we would like to thank the reviewer for even providing us with the sketch of a proof that makes our statement more general (for arbitrary kinetics / enzyme costs and pathway length with the only remaining assumption that the metabolite concentrations in the strains are constant). So we followed the suggestions of the reviewer: (1) in the main text we now use the (entire) metabolite concentration vector in the kinetic rate laws, (2) for the main text, we still use the original example (with equal enzyme costs for both steps) to make the example as simple as possible, (3) we added Supplementary Text S1 with the proof for the more general case as already outlined by the reviewer (we mainly changed notation to be consistent with the main text), (4) we give a more precise statement regarding the conditions when DoL can bring an advantage at all.

*#### Remarks Methods*

The Methods-section is clear and covers all prerequisite knowledge without becoming excessively long or boring. The pseudocode is written very clearly, and will definitely help potential users of ASTHERISC. Several strong assumptions are made in the Methods section, and I feel the following should at least be justified a lot more:

\##### Equation (3): Nr=0. Of course, this seems like a standard assumption that is always made in stoichiometric modelling. However, in this paper the authors are considering the thermodynamic effects that in part depend on metabolite concentrations. The correct formula to use is $Nr=\mu c$, where $\mu$ is the growth rate, and $c$ is the vector of metabolite concentrations. This dilution term implies that all metabolites should have some net production when the growth rate is positive. However, is this small net production still thermodynamically feasible in the solutions that ASTHERISC finds? ASTHERISC might just select a really high metabolite concentration, and it will detect no problem because there does not have to be any net production of this metabolite. I wonder if the authors foresee any problems, or if this issue is negligible. This might be important to add in the text. It is true that this issue is resolved by the following assumption that the growth rate is 0, but this might just be the issue: the dilution of metabolites is one of the reasons that I would say it is unreasonable to set the growth rates to 0.

We thank the reviewer for raising this issue. We agree that, in the case of growth and thus of dilution of metabolites, a net production of those metabolites would be necessary in order to sustain those metabolite concentrations. However, as the reviewer correctly mentioned here and in the following issue, we set the growth rate to zero, mainly because we aim to provide a general feasibility study of partitioning a metabolic production pathway to increase its thermodynamic feasibility (independent of growth). Hence, we focused on the pure production process*.* Clearly, in reality, growth (and thus the dilution and necessary replenishments of metabolite pools) would be needed to be considered when the goal is to have simultaneous biomass and product synthesis. However, another situation could be to decouple growth and production in a two-stage process where no growth occurs in the (second) production phase and where production is initiated (e.g. by dynamic metabolic regulation using temperature- or oxygen-dependent switches). We now discuss this issue (feasibility of growth, two-stage process as a suitable approach etc.) in the Discussion section.

\##### The growth rate of all strains is 0. Now this is a rather strong assumption to make, and, as a reader, I really need to be convinced that this is justified. For example, if ASTHERISC assumes that biomass production is zero, how do I know that the algorithm doesn't choose a division of reactions that makes biomass production impossible for one of the strains? For example, it might be thermodynamically ideal to have a high concentration of ATP in one strain, and a low concentration in another. But can the strain with low ATP still grow? Do the biomass-producing reactions not become thermodynamically infeasible? When an organism is unable to grow, is the chosen division of labor still useful for biotechnological applications? My suggestion is that the authors explicitly state these type of problems. Finding all possible Divisons of Labors is still useful, if they can afterwards be tested for their feasibility. For example, testing if growth is still possible, and if the suggested metabolite exchanges are possible (as the authors do point out in the Dicussion). I would like to see for which multi-strain solutions presented in the paper, growth at these metabolite concentrations is still possible.

This is related to the previous point and we refer to our response to this issue above. Again, the main intention of our manuscript is to provide a generic principle of how production pathways may be split via different strains to improve thermodynamic driving force. Nevertheless, we agree that we need to discuss consequences when feasibility of growth is taken into account (which, as mentioned above, is not necessary in a two-stage process) and added an extended paragraph on this issue in the Discussion section.

\##### All strains have identical fractions. Just like in the example in the Introduction, this assumption feels very limiting, but it might not be. Maybe the authors can justify this assumption, but this is not done at the moment. Now, the reader has to think about the consequences of this assumption, and I think that this is actually a task for the authors. My suggestion is that the authors could point out that they focus on the thermodynamic feasibility of a pathway, and not on resource-allocation. Maybe, the multiplication of all reaction rates of one strain is therefore allowed, and then the biomass fraction of the strain does not matter. To get a feel for the consequences of this assumption, I suggest that the authors write down the full community model without assuming identical fractions (possibly in the SI). Then, the authors may look at the consequences of multiplying the biomass fraction of strain $i$ with a scalar: $F_i \rightarrow \lambda_i F_i$. It will turn out that to keep statisfying the steady-state constraint, all reaction rates of strain $i$ must then be divided by $\lambda_i$. It can then be seen where this will have consequences. I suspect the most important influence in on *satisfying the inhomogeneous flux bounds*. The authors may then explain why this is acceptable. Probably, the authors can argue that leaving this flexibility of choosing the biomass fractions out only takes away flexibility from the model. So, something that is thermodynamically feasible in this constrained case, will also be feasible in the general case. This seems to be true, as long as the authors do not consider kinetics and limited cellular resources.

We thank the reviewer for raising this point. We agree that the fixed fractions may, in general, pose restrictions on the possible solutions. However, except for the total community substrate uptake rate (which is independent from the single-species fractions), the reactions bounds in the used models were free for all reactions (i.e., either [-inf;inf], [-inf;0] or [0;inf]). Together with the fact that we did not consider growth, this implies that the fractions do not pose any restriction in our calculations (and that the found solutions are all scalable). However, in principle, zero-growth models with specific fractions can be easily created with CommModelPy if required for certain calculations. Nevertheless, we also added a brief note on this issue in the Methods section:

“… in this study, we focus on optimal partitioning of the product synthesis pathway (without growth) and the only flux bound used will be the (community) uptake rate of the carbon source. With that we can disregard the production of biomass and assume identical fractions for all involved strain (e.g., 50% in two-strain communities and 33% in three-strain communities). However, in general, growth or/and different biomass fractions may be considered if this is desired. “

*#### Remarks Results:* The results of applying ASTHERISC to example models are presented. The models are well-chosen to illustrate the workings of the model, and the results are exposed in a clear and un-assuming fashion. The example of the community solution serves its purpose: it illustrates the principle underlying ASTHERISC, and its complexity shows why these results cannot be obtained without a computational toolbox.

\##### Page 15: "Reactions for which no $\Delta_rG'^0$ could be found or calculated (93 reactions (18%) in EColiCore2 and 646 (24%) in iML1515) were blocked in the model (rate fixed to zero)" There are several things I'm wondering about after reading this comment. Why is the chosen solution of blocking these reactions the most reasonable? What are the consequences? Is there any particular property of these reactions which explains why the standard Gibbs free energy cannot be found or calculated?

Indeed, as proposed by this reviewer and reviewer 1, in the first ASTHERISC runs we allowed reactions with unknown dG’0 to be used by assuming a free (infinitely negative) dG’0. As a result, ASTHERISC returned many solutions, which often used the reactions with the free dG’0 values with partially very unfavorable (unlikely) metabolite concentrations (e.g., very low substrate concentrations and very high product concentrations for those reactions). This implies that these reactions would have extremely low (negative) dG’0 values. Hence, in these cases, the network gets thermodynamic driving force for free. In fact, flux distributions may arise which are thermodynamically infeasible (e.g., including a cycle) just because the dG’0 of one participating reaction is not specified. Since this manuscript aims to present the general idea of thermodynamics-based community design and because, fortunately, the reactions without dG’0 were generally not part of central metabolic pathways, we decided to block these reactions in order to get consistent results, albeit in a reduced network. We also tested a simplified and naive way of giving all the relevant reactions a dG’0 of 0 kJ/mol, but, again, several returned ASTHERISC solutions relied on those reactions and appeared unrealistic or even thermodynamically infeasible.

Regarding the question of the property of these reactions, we found that the dG’0 is usually not calculable by the eQuilibrator API if at least one the associated metabolites has no representation in the chemical database used by the API.

\##### Bottom page 15: "Interestingly, the highest MDF improvements occur when high product yields are demanded. This indicates that low-yield pathways in the single strain might have high MDFs which are more difficult to improve by a community solution." I agree that this is an interesting note.

We thank the reviewer for this positive note.

\##### Examplary community solution. The authors state that "kdo8p is of interest as its synthase (KDOPS) has been evaluated as a potential antibiotics target". This does not sufficiently explain to me why kdo8p is of interest. How can the over-production of kdo8p be used to inhibit its synthase? It is not that I do not believe that the authors are correct, I just need a little more explanation.

We agree with the reviewer that the meaning of “interest” is not clear in the given context. Instead, this note should just show why kdo8p could be known to the reader. Therefore, we changed the sentence to: “The last step of kdo8p synthesis is catalyzed by the kdo8p synthase (KDOPS), which has been evaluated as a potential antibiotics target.”

I don't understand why the concentration of dha is decreased in ecoli2. The authors state that it is used to facilitate the (reverse) PGI, G6PDH2r and PGL reactions, but I don't see this. dha seems to only be used as a substrate in ecoli2, so why would it not be favourable to maximise its concentration, so that g3p-concentration can be decreased even more. Similarly, one would expect dha to be decreased in ecoli1.

As a first point to answer this question, dha is connected with F6PA (in both ecoli1 and ecoli2), but not with PGI, G6PDH2r or PGL. As a second point, dha is downregulated in ecoli2 in order to enable the exchange of dha from ecoli1 to ecoli2. Due to the negative dG’0 of F6PA, the dha downregulation does not have an impact on the OptMDF.
We clarified the respective sentence, which now reads "Due to the very negative dG’0 of the reverse F6PA reaction, low concentrations of dha and g3p can be used in ecoli2, the latter of which results in a more negative dG’0 of the FBA and TPI reactions.“

\#### Remarks Discussion The discussion is clear and addresses some important points. Especially the paragraph starting with "The application of our algorithm to a metabolic core and a genome-scale model" is very important. Some additional questions that I had with the eventual application of the algorithm:

- I agree that reactions can be knocked-out, but will the optimisation of metabolite concentrations happen automatically? The genetically altered organism has not been evolved to use its new reaction set efficiently, so how do we know that the metabolite concentrations that make the production pathway thermodynamically feasible will be selected?

We agree that there is no guarantee that the metabolite concentrations reach the values that are actually needed to obtain the optimal MDF value (but note that in many cases there are larger concentration ranges for most metabolites under which the optimal MDF value can be reached). We added a note on this issue in the Discussion section:

“… reaching the computed maximal thermodynamic driving forces for the communities also demands that the metabolite concentrations are within the respective ranges required for the optimal MDF and it is not per se clear whether this will be the case or can be adjusted by suitable selection mechanisms.”

- Is there a way to add the energy expenditure needed for exchanging metabolites into the algorithm? I guess, maybe naively, that when a metabolite is exported from a low concentration, and imported to a high concentration, at least one of the transport steps needs to use energy. This means that it should at least be thermodynamically feasible for this strain to produce energy.

Indeed, as also mentioned in the response to reviewer 1, we used a dG’0 of 0 kJ/mol for the (artificial) exchange reactions between the organism and the exchange compartment. This implies that metabolites can only be transported from high to low concentrations (otherwise, a negative MDF would follow and this is excluded for the community solution. Regarding energetic costs (e.g., ATP or proton symport/antiport), these could be taken into account by adding those costs to the respective transport reactions. As demanded by reviewer 1, we tested how the results change when we demand an energetic cost of 1/3 ATP for each transport reaction and found that the results do not change largely (see detailed response to reviewer 1).

- Is there a way in ASTHERISC to select the metabolites that may be exchanged by the algorithm? This does not seem hard to implement.

Yes, the ASTHERISC package already allows one to select the exchangeable metabolites. We added this information in the manuscript in the Methods section: “The standard as well as the extra exchanges can be specifically defined for every application.”

In addition, I would like a discussion of the kinetic analog of this thermodynamic problem. Except for making pathways thermodynamically feasible, Divison of Labor could also help with the efficient usage of cellular resources. Indeed, as the authors show in the Introduction, not when the metabolite concentrations are unchanged, but what if these concentrations are changed. Can this problem also be addressed? If not, why not? Or is it just less important according to the authors?

We thank the reviewer for raising this issue. Indeed, community design could also be based on optimal resource allocation or/and on kinetic rate laws. We think that this is out of scope of this study but see that as a very interesting aspect for future work. We added .a longer paragraph for this issue at the end of the Discussion section.

\#### Minor comments: **Figure 1** I think Figure 1 is rather messy. A reader that did not understand the explanation in the text, will probably not be aided by this figure a lot. This is a matter of taste though, not of right and wrong.

We thank the reviewer for this comment. In principle, we agree that the figure actually has a high information density. However, we would like to keep it as it is as it contains all information needed to understand the limitations of classical DoL.

**Caption Figure 2** Add comma: "would follow, indicating"

Thanks, corrected.

**After Equation 5** Sentence starting with "Furthermore, the biomass fractions" is pretty hard to read.

We thank the reviewer for this comment, and divided the sentence in two parts to make it easier to read.“

**Equation 9** It is not immediately clear whether the $i$-th column is taken before or after the transpose

We thank the reviewer for this comment and changed the corresponding equation and the following one in a more explicit manner.

**Equation 10** I fail to see why the notation is different between Equations (9) and (10): $\Delta_r G_i' \rightarrow \Delta G_i$ and $\hat N \rightarrow N$. These might be typos. If not, more explanation might be needed.

Eq. (10) is only there to make clear that the driving force f_i is the negative of dG’. However we shortened the equation to not repeat the entire equation (9) again.

**Page 11: "A specified minimal fraction of this maximum yield must be reached in all subsequent community model calculations"** Can this fraction also be chosen larger than 1? Or is the maximum possible yield determined without taking into account the thermodynamic feasibility? If so, please indicate this in the text.

Yes, the maximal possible yield is determined without thermodynamic constraints (and thus really presents a true maximum for all (single-species and community) solutions). Fractions greater than 1 are not possible as the given problem would automatically become infeasible. As suggested by the reviewer, we explicitly included this information in the manuscript.

**Page 14** Typo in: "had to be separated in to strains"

Thanks, corrected.

**Page 15** Typo: "For each of the producible target metabolite"

Thanks, corrected.

**Page 17** "Some of these dominant MDF values occurred in all analyzed models." So, there are MDF values that appear often in the core- and in the genome-scale model? If so, it might be interesting to explain a bit more why this is.

We agree with the reviewer that this is an interesting finding, and added a brief discussion in the Results section (paragraph before discussing a specific community solution).

**The use of "exemplary"** It might be that I'm wrong here, but it seems that the word 'exemplary' is used meaning that something is an example, but my interpretation of this word is that it is "a perfect example". It therefore seems a bit like bragging to me when this word is used.

We thank the reviewer for this note, and to avoid a misunderstanding we replaced this word in its occurrences.

**Exemplary community solution** "In the community solution found by ASTHERISC, the MDF value can be increased by 26 % up to 2.87 kJ/mol reaching a yield of 0.733 mol/mol" It confuses me that this yield is here repeated, it seems like this yield is different than the yield of the single-strain model. But this was exactly the yield that was requested, right? So, the reported MDF value for the single-strain model also attains this yield?

Actually, looking at more digits after the decimal point, the reached yield in the example is 0.73311 mol/mol, which is minimally higher than the demanded minimal yield of 98%*0.74775=0.732795 [mol/mol]. Therefore, we added the yield in order to highlight this aspect of this solution since a solution can also reach a much higher percentage of the minimum demanded yield. We added an extended comment on the yield in the manuscript by adding more digits after the decimal mark for the maximal and the reached yield and by extending the referenced sentence, which now reads “In the community solution found by ASTHERISC, the MDF value can be increased by 26% up to 2.87 kJ/mol reaching a yield of 0.73311 mol/mol, which is just 0.04% higher than the demanded minimal yield.”.

**Reviewer #3**

Currently, investigations of mixed synthetic cultures is in the focus of experimental and theoretical studies in biotechnology. Mixed cultures have certain advantages over single species cultivation (for example division of labour) and therefore, the potential of this approach becomes important for the design of processes in various applications. Synthetic mixed cultures could be seen as a mixture of two different species or a mixture of the same species with defined mutations. The manuscript is based on the second variant and analysis the steady-state behaviour of two or three different cultures form a thermodynamic point of view; while standard tools for analysis of metabolic networks like FBA (and its extensions) usually determine intracellular fluxes, more sophisticated tools take into account thermodynamic limitations only for single species. Bekiaris and Klamt ask on a meaningful division of the metabolic network such, that the production of selected compounds of interest is improved (or even first possible) by using more than one strain variant. The criterion used is the MDF, that is, the Max-min-driving force (the maximization of the minimal negative value of the Gibbs energy of each reaction of the entire network/ pathway). The study presented by Bekiaris and Klamt is accurate and comprehensive, and I only have a minor number of comments to improve the manuscript.

We thank the reviewer for his positive view on our manuscript.

(i) How can the minimal OptMDF advantage (0,2 kJ/mol) be interpreted? Are there any reasons for taking just this value?

We chose 0.2 kJ/mol to ensure that the found community solutions provide a minimum gain of thermodynamic driving force compared to a single-strain solution and thus to exclude solutions with only marginal improvements. The value was chosen ad hoc, we felt that, relative to typical MDF values, this is a good choice for a demanded minimal improvement.

(ii) Are there any constraints on the uptake rate of glucose and other main components from the environment?

As described in the Supplementary Text S2 (and now also in the manuscript), we fixed the glucose uptake rate to 1 mmol/(gDW*h). We agree that this is an important information and added it also the main text. Importantly, since no other relevant flux bounds (except –inf, 0, and inf) were considered, the choice of the glucose uptake rate is irrelevant and any found solution can be scaled with an arbitrary positive factor. We chose 1 mmol/(gDW*h) just to ensure that the solutions are normalized with respect to sugar uptake.

(iii) The authors have chosen models with a higher number of reactions and components. For many applications core models are applied? How would the results look like if a standard core model (for example from the COBRA toolbox or the compressed version of the Ecolicor2 with only 82 reactions and 54 metabolites) is used? This is of special interest in light of the result that most exchange metabolites are found in glycolysis or in pathways closely related to glycolysis.

We thank the reviewer for this suggestion. In fact, our intention to use a smaller core (*EColiCore2*; ~500 reactions) as well as a genome-scale (*i*ML1515; ~2700 reactions) model was to compare the solutions from models of different sizes in order to identify recurring patterns and to find out whether the extended set of reactions in the genome-scale model brings out many more (and qualitatively distinct) solutions.

Following the reviewer’s suggestion, we also created a ASTHERISC-compatible version of the *compressed* *EcoliCore2* model (82 reactions compared to the 500 reactions in the uncompressed *EColiCore2* model already used in our study) and successfully ran all 24 scenario calculations on it. We found similar but slightly varying results compared to *ecolicore2double* (e.g., w.r.t. to percentage of MDF advantages by community solutions). The specific solutions, as expected by the reviewer, were more focused on the central carbon metabolism since alternative pathways are not part of this reduced core model. However, we would like to avoid to add even more numbers to the Tables and we also think that the already used (uncompressed) EColiCore2 model can already be seen as a core model and includes also important metabolites such as amino acids (not contained anymore in the compressed EColiCore2 model).

(iv) A basic assumption is that the growth rate for all species is the same. I miss a discussion of the influence of the growth rate on the results. What is the value of the growth rate for the example presented in detail (kdo8p)? Strain ecoli1 takes up glucose and I expect a high growth rate, and I wonder why - in practice - a substrate like dha should be taken up by ecoli2 even in the case a transport system is available? Is it implemented that the growth rate must be equal for both strains (maybe I missed this constrain in the Method section)?

The reviewer is right that, in community models, the growth rate must be identical for all species of the community (this is also mentioned at the beginning of the Methods section, see paragraph after eq. (5)). However, as also mentioned in the Methods section, in all calculations, we fixed the growth rate to 0 for all strains. We chose zero growth in order to focus the solutions on the thermodynamics-based partition of the pathway for product synthesis. Clearly, in reality, growth would be needed to be considered when the goal is to have simultaneous biomass and product synthesis. However, another situation could be to decouple growth and production in a two-stage process where no growth occurs in the (second) production phase and where production is initiated (e.g. by dynamic metabolic regulation using temperature- or oxygen-dependent switches). Since our study is meant to be a general feasibility study of partitioning a pathway to increase its thermodynamic feasibility, we focused on the pure production process*.*

However, since similar aspects were discussed by the other two reviewers, we included a larger paragraph in the Discussion section to discuss this issue

Minor comment: while I could reproduce the value of Figure 2 right, I found a different value for Figure 2 left (-0,245 instead of -0,19).

We thank the reviewer for raising this issue. However, we could reproduce the value of -0.19 and not of -0.245 with our scripts. The reviewer may compare his solution with our solution given in the Supplementary Text S3 (where also all metabolite concentration ranges are given). The script of the toy model run can also be found in the ASTHERISC GitHub repository under the “toy_model” subfolder.
